# Diversity and Ginsenoside Biotransformation Potential of Cultivable Endophytic Fungi Associated With *Panax bipinnatifidus* var. *bipinnatifidus* in Qinling Mountains, China

**DOI:** 10.3389/fphar.2022.762862

**Published:** 2022-04-04

**Authors:** Chao An, Saijian Ma, Xinwei Shi, Chen Liu, Hao Ding, Wenjiao Xue

**Affiliations:** ^1^ Shaanxi Institute of Microbiology, Xi’an, China; ^2^ Engineering Center of QinLing Mountains Natural Products, Shaanxi Academy of Sciences, Xi’an, China; ^3^ Xi’an Botanical Garden of Shaanxi Province, Institute of Botany of Shaanxi Province, Xi’an, China

**Keywords:** *P. bipinnatifidus*, endophytic fungi, diversity, ginsenoside, biotransformation

## Abstract

To obtain novel fungi with potent β-glucosidase for minor ginsenoside production, *Panax bipinnatifidus* var. *bipinnatifidus*, which is a traditional medicinal plant containing various ginsenosides, was first employed to isolate endophytic fungi in this study. A total of 93 representative morphotype strains were isolated and identified according to ITS rDNA sequence analyses, and they were grouped into three phyla (Ascomycota, Basidiomycota, and Mucoromycota), five classes (Dothideomycetes, Sordariomycetes, Eurotiomycetes, Agaricomycetes, and Mucoromycetes), and 24 genera. *Plectosphaerella* (RA, 19.35%) was the most abundant genus, followed by *Paraphoma* (RA, 11.83%) and *Fusariu*m (RA, 9.70%). The species richness index (S, 34) and the Shannon–Wiener index (*H*’, 3.004) indicated that *P. bipinnatifidus* harbored abundant fungal resources. A total of 26 endophytic fungal ethyl acetate extracts exhibited inhibitory activities against at least one pathogenic bacterium or fungus. In total, 11 strains showed strong β-glucosidase activities and also presented with the ability of ginsenoside biotransformation with varied glycoside-hydrolyzing pathways. Excitingly, three genera, namely, *Ilyonectria*, *Sarocladium*, and *Lecanicillium*, and all 11 taxa were first found to have the ability to transform ginsenosides in our study. The results indicated that *P. bipinnatifidus* could be a new fungi resource with potential novel natural compounds with antimicrobial activity and potent β-glucosidase for varied minor ginsenoside production.

## Introduction

Ginseng, the root of *Panax ginseng* C. A. Meyer (Araliaceae), is called the king of all herbs in Asian countries for its various medicinal functions such as anticancer, blood vessel softening, antidiabetic, immunomodulatory, and antiaging effects (Ratan et al., 2021)*.* It is well known that ginsenosides are the most valued and major active components in ginseng. Ginsenosides were consumed worldwide with over two billion USD worth in 2018, and future market demand is expected to be up to trillion USD by 2050 in pharmaceutical, cosmetic, and food industries (Li and Fan, 2020; Yao et al., 2021).

Until now, over 200 ginsenosides have been identified including major and minor ginsenosides. Major ginsenosides, including Rb1, Rb2, Rc, Rd, Re, and Rg1, account for more than 80% of the total ginsenosides in wild ginseng (Kim et al., 2022). However, deglycosylated minor ginsenosides, which are present in very low amounts in most natural ginseng plants, are generally more pharmaceutically active than major glycosylated ginsenosides because of higher bioavailability (Cui et al., 2016; Yang et al., 2021). Therefore, converting major ginsenosides to minor ginsenosides attracted lots of attention (Li and Fan, 2020; Siddiqi et al., 2021; Kim et al., 2022), and several preparations of minor ginsenosides were successfully commercialized with high price due to inefficient production (Li and Fan, 2020). The microbial fermentation and application of the isolated enzymes are the most feasible methods to solve the bottleneck problems because biocatalytic reactions have high substrate specificity, take place under mild conditions, produce just a few byproducts, and require simpler post-processing procedures (Li and Fan, 2020). Therefore, a reservoir of efficient enzymes or microorganisms for different minor ginsenoside production is an urgent requirement.

Endophytes generally inhabit healthy plant tissues without causing disease or injury to the host (Jia et al., 2016). Due to their special living environment and long term coexistence with their hosts, endophytes could produce diverse novel metabolites and are able to incorporate genetic information on bioactive substance biosynthesis from their host plants (Vasundhara et al., 2016). Therefore, endophytes have been widely considered valued resources of natural bioactive compounds and enzymes (Jia et al., 2016; Deshmukh et al., 2018; Gupta et al., 2020). However, considering the immense potential of endophytes for biotransformation of region- and stereo-selective production has remained largely unexplored, although the application of endophytes to biotransformation has been studied for several natural compound conversions such as favans (Agusta et al., 2005), alkaloids (Shibuya et al., 2003), and saponin (Cui et al., 2016; Cheng et al., 2021).

To obtain novel fungi with potent β-glucosidase for minor ginsenoside production, *Panax bipinnatifidus* var. *bipinnatifidus*, which is a traditional medicinal plant containing various ginsenosides and thus was supposed to harbor rich ginsenoside glucosidase resource, was first employed to isolate endophytic fungi in this study. Ginseng total saponins were used as substrates to examine the ginsenoside biotransformation potential of the isolated fungi. Also, antimicrobial activities of extracts of the fungal fermentation broth were determined. To the best of our knowledge, this is the first report on the diversity, antimicrobial, and ginsenoside biotransformation activities of cultivable endophytic fungi associated with *P. bipinnatifidus*.

## Materials and Methods

### Materials

Ginsenoside extracts from the roots of *Panax ginseng* C. A. Meyer. (saponin contents > 67%) and standard ginsenosides Rb1, Rb2, Rc, Rd, Re, F2, Rg3, Rh2, and Compound K (CK) were purchased from Chengdu DeSiTe Biological Technology Co., Ltd. Methanol and acetonitrile (HPLC grade) were obtained from Merck (Darmstadt, Germany). All other reagents and chemicals were at least of analytical grade.

### Plant Collection and Isolation of Fungal Endophytes

In August 2020, wild and healthy *P. bipinnatifidus* samples were randomly collected from Yingpan town, Shaanxi province of China (33°48′21″ N, 108°56′23″ E, elevation, 1165 m), and were identified by associate researcher XinWei Shi at the Xi’an Botanical Garden of Shaanxi Province. These plants were carefully uprooted from their native habitats and placed in a sampling bag, which were immediately transported to the laboratory and stored in a refrigerator (4°C), as described previously (Tan et al., 2014). The endophytic fungal isolation procedure was carried out within 24 h of sample collection. The plant tissues were pretreated with the protocol mentioned by Qin et al. (2019). In brief, fifteen root tissues from healthy plants were washed sequentially in running tap water, ultrasonic bath (200 W, 10 min), and dried naturally at room temperature. Subsequently, the plant tissues were surface-sterilized with the method described by Tan et al. (2014) with minor modifications. In brief, the roots tissues were disinfected by sequential immersion in 70% ethanol for 1 min, 2.5% NaClO_2_ for 3 min, and 70% ethanol for 1 min. A total of 150 fragments (approximately 0.3 cm long and 0.3 cm wide) were excised from the pretreatment plant tissues. Tissue segments were plated on a PDA medium supplemented with amikacin sulfate (100 U/mL). The plates (90 mm) placed with seven segments were incubated at 28°C for 1 week. Emergent fungal colonies were re-cultured and photographed in a PDA medium. Finally, the pure isolates were stored at −80°C in a 20% glycerol solution in the Engineering Center of QinLing Mountains Natural Products, Shaanxi Provincial Institute of Microbiology.

### Molecular Identification and Phylogenetic Analyses

The mycelia of endophytic fungi, which were purified and cultured on a PDA medium plate at 28°C for 7 days, were pulverized in liquid nitrogen with a mortar and pestle. The pretreated samples were used for the extraction of genomic DNA using the TaKaRa MiniBEST Bacterial Genomic DNA Extraction Kit (Dalian, China). Internal transcribed spacer (ITS) sequence amplification was conducted using the universal primers ITS1 (5′-TCC​GTA​GGT​GAA​CCT​GCG​G-3′) and ITS4 (5′-TCC​TCC​GCT​TAT​TGA​TAT​GC-3′), according to the description by White et al. (1990) with minor modifications. Each 50 μl final reaction mixture contained 5.0 μL 10×Taq buffers, 4.0 μL 200 mmol/L dNTPs, 2.0 μL each primer (10 μM), 0.5 μL Ex Taq enzyme (TaKaRa, Dalian), 5.0 μL genomic DNA, and 31.5 μL ddH_2_O. The PCR assay was performed in a TProfessional Standard 96 Gradient thermal cycler (Biometra, Jena, Germany) programmed at 95°C for 5 min, 35 cycles at 95°C for 30 s, 55°C for 30 s, 72°C for 1 min, and 72°C for 10 min. 5 μL of each PCR product was analyzed electrophoretically in 1% (w/v) agarose gels stained with GelRed (Shanghai Generay Biotech Co., Ltd., China). All positive PCR products were subsequently purified and sequenced by BGI Biotechnology (Shenzheng, China). The aligned and edited sequences with MEGA 5.05 (Arizona State University, Tempe, United States) were used to sequence analysis in the National Center for Biotechnology Information (NCBI) GenBank database (http://www.ncbi.nlm.nih.gov/) and match similar sequences. The sequence with similarity over 97% is considered to be in the same genus. The evolutionary history was inferred, as described by Wei et al. (2018) and Felsenstein (1985). All sequences, which are aligned by MEGA 5.05 using Clustal W (Arizona State University, Tempe, United States) and deleted all positions containing gaps, were used to construct the maximum likelihood phylogenetic trees by MEGA software 5.05 (Stamatakis, 2006).

### Crude Extract Preparation of Fungal Fermentation Broth

Five agar plugs (9 mm diameter) of each fungus, which were cultured on the PDA for 7 days, were inoculated in 250 ml flasks containing 50 ml of potato dextrose broth (PDB) (200 potatoes (peeled potatoes, cut into 1 cm pieces, boiled for 20 mins, and filtered to obtain filtrate), 20 dextrose, pH 6.0) and placed in a rotary shaker at 28°C and 230 rpm for 14 days. The fermentation broth was centrifuged at 8000 rpm for 8 min. 50 mL of the culture filtrate was extracted three times with an equal volume ethyl acetate, and the total organic phase was separated using a rotary evaporator in a 50°C water bath, as described by Xing et al. (2011) with minor modifications. The residue was weighed and diluted in pure methanol (10 mg/ml final concentration) and sterilized by filtration using an organic filter (0.22 μm, Shanyu Co., Ltd., China).

### Antimicrobial Activity

Seven pathogens such as *Bacillus cereus*, *Escherichia coli*, *Bacillus subtilis*, *Staphylococcus aureus*, *Pseudomonas aeruginosa*, *Xanthomonas oryzae* pv*. oryzae*, and *Candida albicans* were used as test organisms to evaluate the antimicrobial activities of the ethyl acetate crude extracts from culture filtrates of 93 fungal strains using the agar diffusion protocol mentioned by Wang et al. (2019a). Precisely, the sterilized medium mixed with pre-cultured pathogens was poured into a Petri dish (90 mm) and allowed for solidification, and the agar blocks were removed with a sterilized toothpick. *C. albicans* was grown in Sabouraud agar medium at 28°C, and the other bacteria were grown in the Luria Bertani agar (LB) medium at 37°C. Concentrations of 10 mg/mLfor EtOAc extracts were added to the hole of the test plate and incubated at 37°C for 24 h for pathogenic bacteria or for 48 h for *C. albicans*. The aliquot of ampicillin sodium and actidione was dissolved in distilled water to a concentration of 1 mg/ml and used as a positive antimicrobial control. In addition, pure methanol was used as a negative control. Antimicrobial activities were evaluated by measuring the diameter of the inhibition zones. All experiments were replicated three times.

### Screening of β-Glucosidase Producing Endophytic Fungi

The β-glucosidase activities were screened with the method mentioned by Cui et al. (2016). The isolates, which have grown on a PDA plate for 5 days, were inoculated on Esculin-R2A agar. The endophytic fungi with β-glucosidase activities can hydrolyze esculin and appear as colonies surrounded by a reddish-brown to dark brown zone. Esculin-R2A agar contains (g/L) Esculin 1, ferric citrate 0.5 with 15.2 R2A agar medium, and autoclaved at 121°C for 20 min.

### Transformation of Ginseng Total Saponins

The isolates with β-glucosidase activities were inoculated into 250 ml flasks containing 30 ml of fermentation culture described by Ye et al. (2010) with minor modifications (20 g glucose, 10 g yeast powder, 0.5 g NaNO_3_, 1 g KH2PO_4_, 2 g Na_2_HPO_4_, 0.2 g FeSO_4_, 0.1 g ZnSO_4_, 0.2 g CuSO_4_, 1 g CaCl_2_, and 0.5 g MgSO_4_. 7H_2_O in 1 L of distilled water, initial pH of 6.5), and incubated at 28°C on a rotary shaker at 180 rpm. The ginsenoside extracts were first dissolved in methanol at a final concentration of 2 g/L and then supplied in equal volumes into the culture broth, which were incubated with the endophytic fungi and cultivated on a rotary shaker at 180 rpm for 72 h. The fungi were continually cultured for 48 h under these conditions; 2 ml of fungal suspension was aseptically removed from a shake flask culture and centrifuged to remove the precipitate. The residue was dissolved in methanol, and analysis of the biotransformation ability of ginsenosides was carried out by HPLC.

### HPLC Analysis

Nine standard ginsenosides were first prepared in 1 g/L methanol solution, and the mixed standard samples containing nine standard ginsenosides were diluted to 100 mg/L with the methanol solution. Using the HPLC method described by Park et al. (2017) with minor modifications, all samples were quantitatively analyzed by HPLC (Waters, alliance separation module 2695, 2998 detector; Waters, Milford, MA, United States) using a C18 column (YMC-Pack ODS-AQ, 250 mm × 4.6 mm, 5 μm, YMC, Japan) with a column temperature of 30°C. The mobile phase was A (water) and B (acetonitrile). Gradient elution started with 77% solvent A (water) and 23% solvent B (acetonitrile) and was then changed to A from 77% to 48%, 0–12 min; A from 48% to 25%, 12–35 min; A from 48%, 35–36 min; and A from 48% to 77%, 36–42 min. All samples were detected by absorption at 203 nm, with an inject volume of 10 μL.

### Diversity Analyses of the Endophytic Fungi

The isolation frequency (IF) was defined as the frequency of the occurrence of the specific endophytic fungi in total isolates based on the number of isolates (N). The relative abundance (RA) was calculated based on the number of all isolate numbers (N). The diversity of fungal species from *P. bipinnatifidus* was evaluated using the species richness index (S) and Shannon–Weiner index (*H*′), with the protocol mentioned by Fedor and Spellerberg (2013). The species richness index (S) was the number of endophytic fungal species obtained from the corresponding plant tissues. A total of 93 isolates were evaluated for the antimicrobial activities in this study.

### Statistical Analyses

All results were expressed as the mean ± SEM. Graphs were prepared by Excel 2010 (Microsoft, United States).

## Results

### Isolation, Sequencing, Identification, and Diversity Analyses of the Endophytic Fungi from *P. bipinnatifidus*

In this study, a total of 93 fungal colonies were successfully isolated from 150 tissue segments of *P. bipinnatifidus* with a potato dextrose agar (PDA) medium. As shown in Table 1, all isolates were grouped into 34 taxa by blasting their ITS rDNA regions using the BLAST in the NCBI GenBank database (Supplementary File S1) and constructing the phylogenetic trees using the maximum likelihood method (Supplementary File S2). The species richness index (S) and Shannon–Wiener index *(H')*, which are two important parameters for diversity analysis, were 34 and 3.004 for *P. bipinnatifidus*, respectively. As presented in Table 2, these isolates have been further grouped into three phyla (Ascomycota, Basidiomycota, and Mucoromycota), five classes (Sordariomycetes, Eurotiomycetes, Dothideomycetes, Mucoromycetes, and Agaricomycetes), 11 orders, 16 families, and 24 fungal genera.

**TABLE 1:** Identification of endophytic fungi from *P. bipinnatifidus* by the Basic Local Alignment Search Tool (BLAST) in the GenBank database.

No	Closest species		Isolate numbers	Identity (%)	N	IF
1	*Alternaria alstroemeriae* CBS 118809 MH863036		F8528	99.43	1	1.08
2	*Aspergillus fumigatus* ATCC 1022 (HQ026746)		F8564, F8570, and F8913	98.94	3	3.23
3	*Aspergillus pseudodeflectus* NRRL 6135 (EF652507)		F8512	98.90	1	1.08
4	*Aspergillus ochraceus* NRRL 398 (AY373856)		F8573	98.93	1	1.08
5	*Aspergillus brunneoviolaceus* NRRL 4912 (EF661220)		F8561	99.62	1	1.08
6	*Aspergillus foetidus* CBS 121.28 (MH854949)		F8563	99.10	1	1.08
7	*Aspergillus terreus* ATCC 1012 (AY373871)		F8915	99.82	1	1.08
8	*Ceratobasidium ramicola* CBS 133.82 (DQ278931)		F8538	89.04	1	1.08
9	*Cladosporium oxysporum* CPC 14371 (HM148118)		F8569	99.22	1	1.08
10	*Colletotrichum kakivorum* KCTC 46679 (LC324781)		F8879	99.61	1	1.08
11	*Dactylonectria alcacerensis*		F8548, F8549, F8500, and F8867	99.61	4	4.30
12	*Daldinia childiae* CBS 122881 (KU683757)		F8571	94.38	1	1.08
13	*Diaporthe ukurunduensis* CFCC 52592 (MH121527)		F8533	95.76	1	1.08
14	*Exserohilum gedarefense* CBS 297.80 (KT265244)		F8515	99.64	1	1.08
15	*Flavodon ambrosius* BPI 893213 (KR119072)		F8914	86.19	1	1.08
16	*Fusarium solani* CBS 140079 (KT313633)		F8504, F8521, F8523, F8524, F8526, F8545, F8558, F8880, and F8916	99.07	9	9.68
17	*Lecanicillium coprophilum* CGMCC 3.18986 (MH177616)		F8542	93.26	1	1.08
18	*Ilyonectria robusta* CBS 308.35 (JF735264)		F8499, F8502, F8532, F8541, F8559, F8868, F8870, and F8874	99.41	8	8.60
19	*Paraphoma radicina* CBS 111.79 (KF251172)		F8501, F8511, F8514, F8536, F8537, F8540, F8544, F8553, F8557, F8862, and F8864	99.41	11	11.83
20	*Penicillium chrysogenum* CBS 306.48 (AY213669)		F8565	98.91	1	1.08
21	*Penicillium rubens* CBS 129667 (JX997057)		F8568	99.63	1	1.08
22	*Penicillium expansum* ATCC 7861 (AY373912)		F8517 and F8566	99.63	2	2.15
23	*Penicillium citrinum* NRRL 1841 (AF033422)		F8560 and F8562	99.41	2	2.15
24	*Penicillium camponotum* CBS 140982 (KT887855)		F8572	98.19	1	1.08
25	*Periconia aquatica* HKAS 92754 (KY794701)		F8503	93.81	1	1.08
26	*Plectosphaerella oligotrophica* CGMCC 3.15078 (JX508810)		F8496, F8497, F8520, F8522, F8525, F8529, F8531, F8535, F8543, F8547, F8550, F8555, F8860, F8865, F8871, F8875, and F8876	98.47	17	18.28
27	*Plectosphaerella niemeijerarum* CBS 143233 (MG386080)		F8866	99.22	1	1.08
28	*Pseudopyrenochaeta terrestris* CBS 282.72 (LT623228)		F8498, F8519, F8530, F8544, F8861, and F8909	94.98	6	6.45
29	*Rhizopus oryzae* CBS 112.07 (EU484274)		F8518, F8546, and F8911	99.51	3	3.23
30	*Rigidoporus macroporus* BJFC 010373 (KT203298)		F8912 and F8917	90.13	2	2.15
31	*Sarocladium bacillisporum* CBS 425.67 (HE608639)		F8513 and F8527	97.22	2	2.15
32	*Talaromyces variabilis* CBS 385.48 (JN899343)		F8516 and F8567	96.69	2	2.15
33	*Thelonectria blackeriella* CBS 142200 (KX778711)		F8863 and F8877	94.41	2	2.15
34	*Volutella delonicis* MFLU 19-1384 (MT215504)		F8551	90.61	1	1.08
Total					93	100
Species richness (S)			34
Shannon–Wiener index (H′)			3.004

**TABLE 2:** Taxa of all endophytic fungi isolates from *P. bipinnatifidus*.

No	Phylum	Class	Order	Family	Genus	Number (N)
1	Ascomycota	Sordariomycetes	Glomerellales	Plectosphaerellaceae	*Plectosphaerella*	18
2			Hypocreales	Cordycipitaceae	*Lecanicillium*	1
3				Sarocladiaceae	*Sarocladium*	2
4				Nectriaceae	*Dactylonectria*	4
5					*Fusarium*	9
6					*Ilyonectria*	8
7					*Thelonectria*	2
8					*Valutella*	1
9			Glomerellales	Glomerellaceae	*Colletotrichum*	1
10			Xylariales	Hypoxylaceae	*Daldinia*	1
11			Diaporthales	Diaporthaceae	*Diaporthe*	1
12			Cladosporiales	Cladosporiaceae	*Cladosporium*	1
13		Eurotiomycetes	Eurotiales	Aspergillaceae	*Aspergillus*	8
14					*Penicillium*	7
15				Trichocomaceae	*Talaromyces*	2
16		Dothideomycetes	Pleosporales	Massarineae	*Periconia*	1
17				Pleosporineae	*Alternaria*	1
18					*Exserohilum*	1
19					*Paraphoma*	11
20					*Pseudopyrenochaeta*	6
21	Mucoromycota	Mucoromycetes	Mucorales	Mucorineae	*Rhizopus*	3
22	Basidiomycota	Agaricomycetes	Cantharellales	Ceratobasidiaceae	*Ceratobasidium*	1
23			Irpicaceae	Irpicaceae	*Flavodon*	1
24				Meripilaceae	*Rigidoporus*	2
Total	3	5	11	16	24	93

### Relative Abundance Analyses of Endophytic Fungi From *P. bipinnatifidus*

**FIGURE 1:**
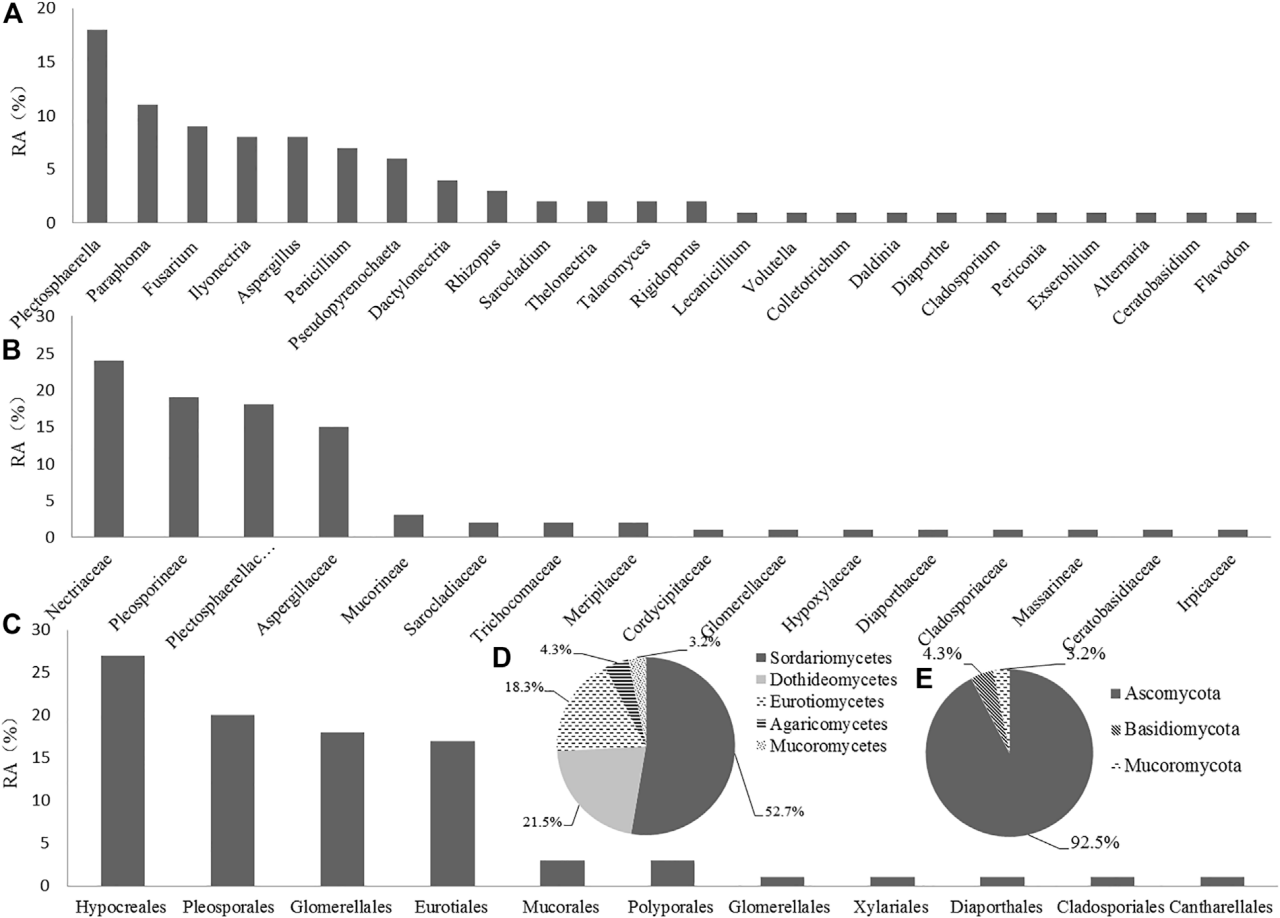
Relative abundance (RA, %) of endophytic fungi at the level of genus **(A)**, family **(B)**, order **(C)**, class **(D)**, and phylum **(E)**.

As shown in Table 1, *Plectosphaerella oligotrophica*, *Paraphoma radicina*, and *Fusarium solani* were dominant species, and their IF were 18.28%, 11.83%, and 9.68%, respectively. The relative abundance (RA) of these isolates at genus, family, order, class, and phylum levels are shown in Figure 1(A–E), respectively. At the genus level, *Plectosphaerella* (RA, 19.35%) was the most abundant, followed by *Paraphoma* (RA, 11.83%) and *Fusarium* (RA, 9.70%). At the family level, Nectriaceae (RA, 25.81%), Pleosporineae (RA, 20.43%), and Plectosphaerellaceae (RA, 19.35%) were the three most abundant groups in this study. At the phylum level, the majority of fungi isolated from *P. bipinnatifidus* were identified as Ascomycota (RA, 92.47%), which represented three classes (Sordariomycetes (RA, 52.69%), Eurotiomycetes (RA, 21.51%), and Dothideomycetes (RA, 18.28%)). In addition, one class (Agaricomycetes, RA, 4.31%) belonged to Basidiomycota (RA, 4.31%), and one class (Mucoromycetes, RA, 3.23%) belonged to Mucoromycota (RA, 3.23%).

### Antimicrobial Activity Screening of the Ethyl Acetate Extracts From Endophytic Fungal Culture Filtrates

As shown in Table 3, 26 out of 93 endophytic fungal ethyl acetate extracts exhibited inhibitory activity against at least one pathogenic bacterium or fungus (Supplementary File S3). The other 67 extracts did not show inhibitory activities. These 26 strains belonged to genera of *Alternaria*, *Aspergillus*, *Cladosporium*, *Dactylonectria*, *Exserohilum*, *Fusarium*, *Ilyonectria*, *Lecanicillium*, *Penicillium*, *Pseudopyrenochaeta*, *Rhizopus*, *Rigidoporus*, *Sarocladium*, and *Talaromyces.*

**TABLE 3:** Antimicrobial activities of culturable endophytic fungi from *P. bipinnatifidus*.

Isolates No	Taxa (accession number)	Inhibition zone in diameter on the Petri plate (mm)						
		*B. cereus*	*E. coli*	*B. subtilis*	*S. aureus*	*P. aeruginosa*	*R. solanacearum*	*C. albicans*
F8498	*Pseudopyrenochaeta terrestris* (MZ572981)	10.77 ± 0.28	—	8.46 ± 0.09	10.19 ± 0.12	—	—	12.20 ± 0.18
F8513	*Sarocladium bacillisporum* (MZ572990)	10.60 ± 0.09	—	10.61 ± 0.36	11.97 ± 0.16	—	—	—
F8515	*Exserohilum gedarefense* (MZ572992)	8.47 ± 0.10	—	8.48 ± 0.05	—	—	—	—
F8519	*Pseudopyrenochaeta terrestris* (MZ572996)	14.01 ± 0.15	—	13.93 ± 0.11	13.27 ± 0.16	—	—	16.20 ± 0.17
F8521	*Fusarium solani* (MZ572998)	8.65 ± 0.14	—	—	—	—	—	—
F8528	*Alternaria alstroemeriae* (MZ573005)	15.57 ± 0.33	10.50 ± 0.30	15.31 ± 0.17	14.48 ± 0.29	10.41 ± 0.37	—	11.63 ± 0.22
F8542	*Lecanicillium coprophilum* (MZ573017)	11.27 ± 0.17	—	12.17 ± 0.16	11.88 ± 0.11	—	—	—
F8549	*Dactylonectria alcacerensis* (MZ573024)	—	—	—	11.85 ± 0.24	—	—	—
F8559	*Ilyonectria robusta* (MZ573031)	11.77 ± 0.23	—	11.07 ± 0.13	14.19 ± 0.30	—	10.12 ± 0.13	—
F8560	*Penicillium citrinum* (MZ573032)	11.35 ± 0.25	—	10.18 ± 0.18	9.26 ± 0.40	—	11.02 ± 0.12	—
F8561	*Aspergillus brunneoviolaceus* (MZ573033)	12.18 ± 0.22	9.03 ± 0.10	11.30 ± 0.16	12.06 ± 0.17	9.00 ± 0.10	15.25 ± 0.24	11.04 ± 0.14
F8562	*Penicillium citrinum* (MZ573034)	12.22 ± 0.21	—	11.84 ± 0.25	11.34 ± 0.21	9.02 ± 0.11	11.54 ± 0.34	—
F8563	*Aspergillus foetidus* (MZ573035)	12.97 ± 0.24	9.17 ± 0.10	12.62 ± 0.21	12.14 ± 0.26	9.89 ± 0.30	11.75 ± 0.27	—
F8564	*Aspergillus fumigatus* (MZ573036)	11.56 ± 0.22	—	9.96 ± 0.26	11.06 ± 0.42	—	10.78 ± 0.20	—
F8565	*Penicillium chrysogenum* (MZ573037)	9.32 ± 0.14	—	9.28 ± 0.27	-	—	—	—
F8566	*Penicillium expansum* (MZ573038)	11.15 ± 0.20	—	10.69 ± 0.35	13.70 ± 0.23	—	—	—
F8567	*Talaromyces variabilis* (MZ573039)	11.56 ± 0.23	—	10.79 ± 0.18	13.85 ± 0.22	—	—	—
F8568	*Penicillium rubens* (MZ573040)	12.61 ± 0.76	—	12.25 ± 0.27	11.78 ± 0.28	—	—	9.63 ± 0.28
F8569	*Cladosporium oxysporum* (MZ573041)	9.05 ± 0.32	—	—	10.14 ± 0.73	—	—	—
F8570	*Aspergillus fumigatus* (MZ573042)	13.93 ± 0.39	—	13.08 ± 0.32	18.27 ± 0.22	—	—	—
F8573	*Aspergillus ochraceus* (MZ573045)	12.03 ± 0.07	—	11.25 ± 0.27	12.22 ± 0.54	8.13 ± 0.20	—	9.32 ± 0.14
F8911	*Rhizopus oryzae* (MZ573065)	8.88 ± 0.33	—	11.49 ± 0.42	11.64 ± 0.26	—	—	—
F8912	*Rigidoporus macroporus* (MZ573066)	31.13 ± 0.73	24.35 ± 0.45	31.30 ± 0.79	30.49 ± 1.05	24.44 ± 0.61	34.81 ± 1.05	11.96 ± 0.35
F8913	*Aspergillus fumigatus* (MZ573067)	12.87 ± 0.36	—	13.16 ± 0.21	17.32 ± 0.03	—	—	—
F8915	*Aspergillus terreus* (MZ573069)	17.91 ± 0.35	—	18.31 ± 0.62	21.33 ± 0.57	11.04 ± 0.12	21.84 ± 0.47	11.11 ± 0.35
F8916	*Fusarium solani* (MZ573070)	31.13 ± 0.44	25.36 ± 0.49	32.87 ± 0.35	32.98 ± 0.23	22.12 ± 0.27	36.91 ± 0.80	12.47 ± 0.10
Positive control-1	Ampicillin sodium	21.32 ± 0.45	15.53 ± 0.32	24.42 ± 0.42	22.46 ± 0.38	18.58 ± 0.35	20.18 ± 0.25	—
Positive control-2	Actidione	—	—	—	—	—	—	22.05 ± 0.47
Negative control	Methanol	—	—	—	—	—	—	—

### Screening β-Glucosidase Activity Producing Endophytic Fungi

**FIGURE 2:**
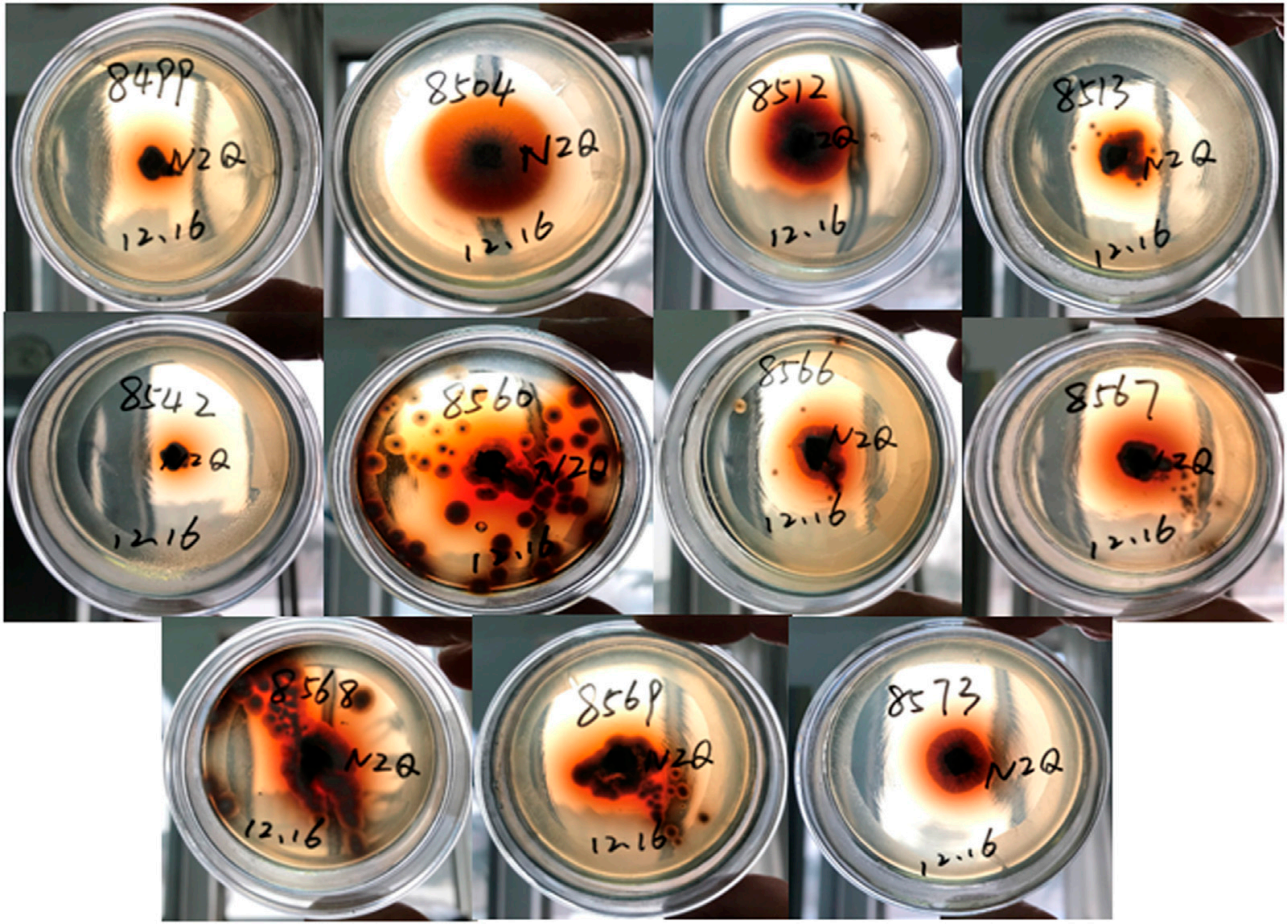
Screening of β-glucosidase-producing endophytic fungi in Esculin-R2A agar.

β-glucosidase activities were preliminarily screened for 93 endophytic fungi on plates using Esculin-R2A agar medium. Among these endophytes, 11 endophytic fungi, which were identified and named F8499 [*Ilyonectria robusta* (MZ572982)], F8504 [*Fusarium solani* (MZ572987)], F8512 [*Aspergillus pseudodeflectus* (MZ572989)], F8515 [*Sarocladium bacillisporum* (MZ572990)], F8542 [*Lecanicillium coprophilum* (MZ573017)], F8560 [*Penicillium citrinum* (MZ573032)], F8566 [*Penicillium expansum* (MZ573038)], F8567 [*Talaromyces variabilis* (MZ573039)], F8568 [*Penicillium rubens* (MZ573040)], F8569 [*Cladosporium oxysporum* (MZ573041)], and F8573 [*Aspergillus ochraceus* (MZ573045)], showed the strongest capacity of β-glucosidase-producing activity (Figure 2).

### Biconversion of Major Ginsenosides From Ginseng Extracts

**FIGURE 3:**
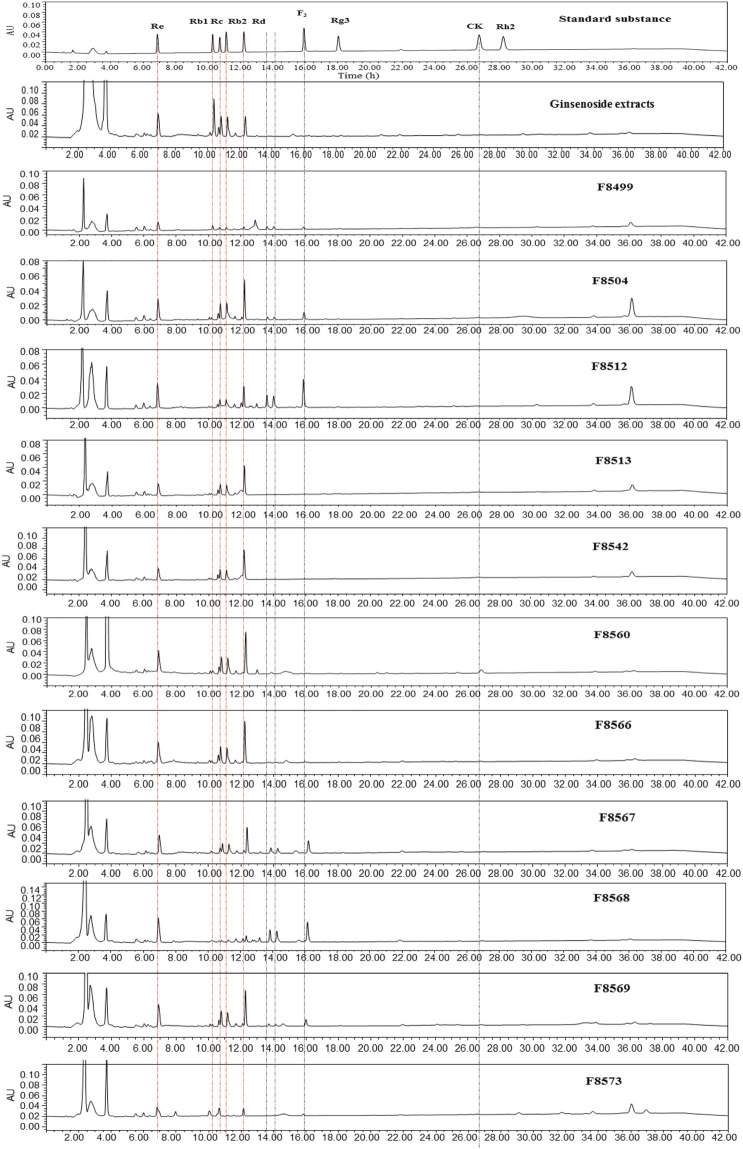
HPLC spectra of the ginsenoside standards and ginsenoside biotransformation products by 11 strains with β-glucosidase activity.

**FIGURE 4:**
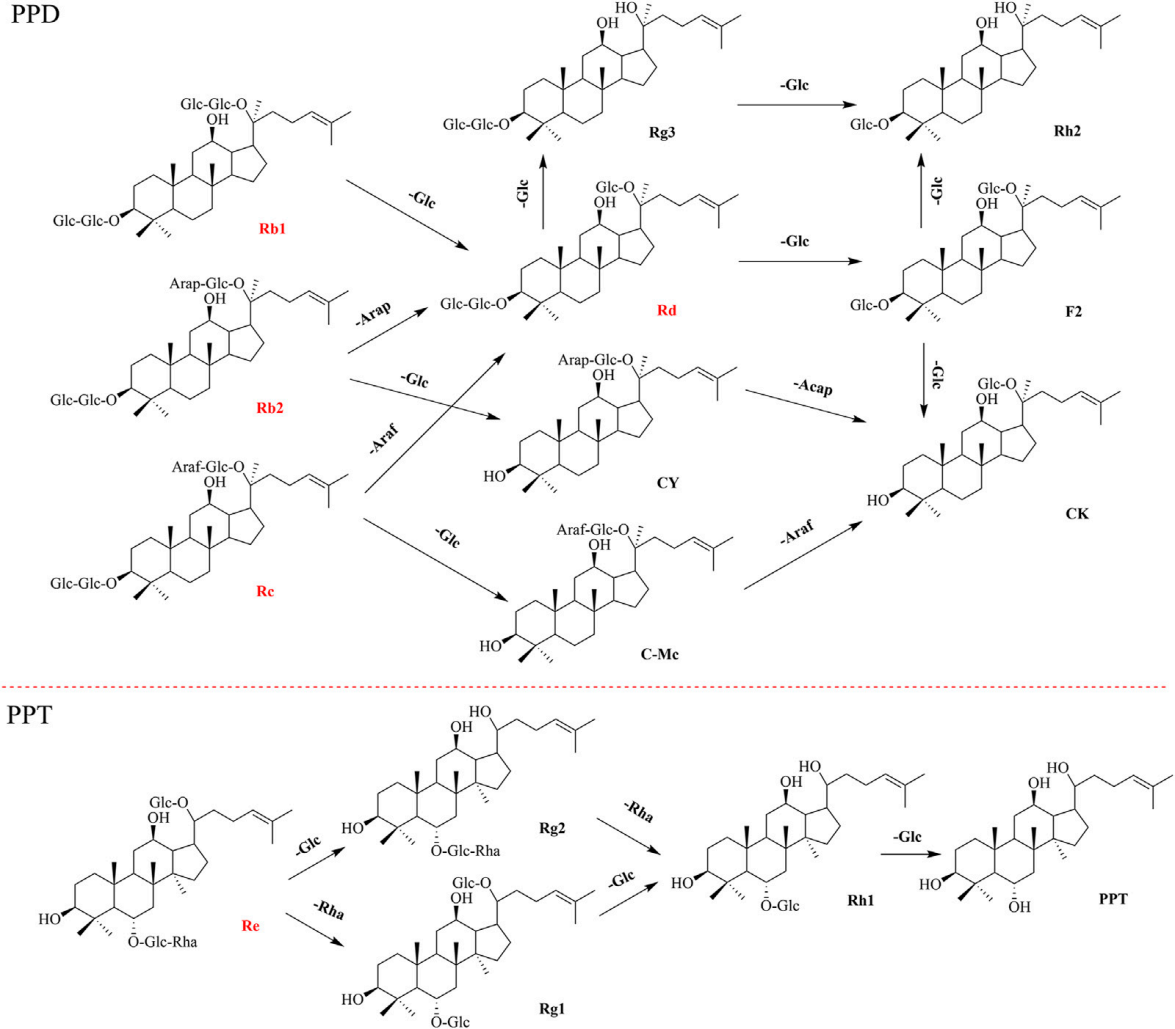
Schematic diagram of directed biotransformation of major ginsenosides (red section) of the extracts from *P. ginseng* roots. PPD (Protopanaxadiol-type ginsenoside) and PPT (Protopanaxatriol-type ginsenoside).

To evaluate the ginsenoside biotransformation ability of these endophytic fungi with β-glucosidase activity, we tested the composition of ginsenoside from fermentation broth before and after cultivating at the shaker level by HPLC methods (Figure 3). The results showed that 1) total saponins from ginseng extracts mainly contained major glycosylated ginsenosides such as Rb1, Rb2, Rc, Re, and Rd; 2) 11 endophytic fungi with β-glucosidase activity showed the capacity to biotransform major glycosylated ginsenosides to Rd, F2, CK, and the other unidentified saponins; and 3) all isolates could convert Rb1 to other ginsenosides, while Rb2 and Rc were transformed by four strains, and Re transformation was obtained by two strains. According to the literature reports (Geraldi, 2020), proposed transformation pathways for the isolates are summarized in Figure 4. Seven strains (F8499, F8504, F8512, F8567, F8568, F8569, and F8573) were able to transform ginsenoside Rb1 to F2 by the transformation pathway as follows: Rb1→Rd→F2; 3 strains (F8513, F8542, and F8566) were only able to transform ginsenoside Rb1 to Rd; F8560 was able to transform major ginsensides Rb1 to minor ginsensides CK directly as the following pathways: Rb1→Rd→F2→CK. Rb2, and Rc can be also transformed by endophytic fungi (F8499, F8567, F8568, F8573), but the transformation products were unidentified due to lack of adequate ginsenoside standards. Ginsenoside Re, which belonged to protopanaxatriol-type ginsenoside, can also be transformed by endophytic fungi (F8499 and F8573).

## Discussion

*Panax bipinnatifidus* var. *bipinnatifidus* (also known as *Panax japonicas* var. *major* (Burkill) C.Y.Wu and Feng *bipinnatifidus*) is a perennial herb mainly distributed in China (Li et al., 2019). The root of *P. bipinnatifidus*, known as Zhuzi (means bead due to its appearance) ginseng locally*,* has been used as traditional medicine for thousands of years in Qinling Mountains, China. Modern pharmacological studies showed that the oleanane-type triterpenoid saponins (also known as ginsenosides) are the main chemical constituents in *P. bipinnatifidus* (Zhu et al., 2004; Tung et al., 2011)*.* Based on the assumption that the endophytes incorporate genetic information on bioactive substance biosynthesis from their host plants, we supposed that endophytes from *P. bipinnatifidus* harbored rich enzyme resource for ginsenoside transformation. However, endophytic fungi resource associated with *P. bipinnatifidus* had remained unexplored.

In this study, 93 representative morphotype strains were isolated and identified from *P. bipinnatifidus*. var. *bipinnatifidus* in Qinling Mountains, China. Further diversity analysis showed that abundant fungal endophyte resources were harbored in this plant with the species richness index (S) of 34 and Shannon–Wiener index *H*′ of 3.004. The isolated fungi taxa also differed from those isolated from the different plants in the same region. In our previous study, it was found that there was some similarity between the endophytic fungi composition of *C. japonicus* Sieb (dominant genera: *Colletotrichum, Aspergillus,* and *Diaporthe*) and that of *T. chinensis* Baker (dominant genera: *Collectotrichum, Fusarium, Aspergillus*) from Yingpan town (Qinling Mountains region), Shaanxi province of China. However, the results obtained in this study (dominant genera: *Plectosphaerella, Paraphoma,* and *Fusarium*) showed significant difference from those of the aforementioned studies (An et al., 2020a; An et al., 2020b). Also, rare genera such as *Lecanicillium*, *Sarocladium*, *Thelonectria*, *Valutella*, *Exserohilum*, *Flavodon*, and *Rigidoporus* obtained in this study were quite different from those in our previous studies, including *Biscogniauxia*, *Leptostroma*, *Stachybotrys*, *Didymella*, *Peniophora*, *Peniophora*, *Rhodotorula*, *Setophaeosphaeria*, *Clitopilus*, *Muyocopron*, *Thanatephorus*, and *Ceriporia*. The result was in accordance with the previous studies that the endophytic fungal community composition was significantly influenced by the host geographic area, habitat environments, and growth cycle (Wang et al., 2019b). It was also suggested that highly abundant endophytic fungi resources remained unexplored in the Qinling Mountain area, which is the most important natural climatic boundary between the subtropical and warm temperate zones of China, and support an astonishingly high biodiversity (Ma et al., 2018) and are rich in medicinal plant resources.

Generally speaking, endophytes represent a wide source of unexplored and uncharacterized microorganisms capable of producing novel metabolites (Deshmukh et al., 2018), although they have been widely recognized as an important source of structurally diverse and pharmacologically active natural products (Katoch and Pull, 2017). Therefore, the isolates from *P. bipinnatifidus* were evaluated for their abilities to produce natural products with antimicrobial activity. The results showed that 26 out of 93 isolates exhibited inhibitory activity against at least one pathogenic bacterium or fungus. Among these, 26 strains (belonged to 14 genera) and seven genera, including *Alternaria*, *Aspergillus*, *Cladosporium*, *Dactylonectria*, *Fusarium*, *Penicillium*, and *Talaromyces*, have been widely reported to produce antimicrobial activities using natural products (Deshmukh et al., 2014; Deshmukh et al., 2018). Nonetheless, new antibiotics were still isolated from these genera of endophytes (Luyen et al., 2019; Qi et al., 2019; Zhang et al., 2021). In addition, the other seven genera such as *Exserohilum*, *Lecanicillium*, *Ilyonectria*, *Rhizopus*, *Rigidoporus*, *Sarocladium*, and *Pseudopyrenochaeta* with antimicrobial activity have rarely been reported to produce antimicrobial substances. Anyway, the results suggested these isolates have a potential for novel metabolite production.

Recent studies revealed that varied ginsenosides showed pharmaceutical potential with different health benefits because of the structural differences, such as the location of glycosides and the sugar type (Kim et al., 2018). Most ginsenosides contain a dammarane skeleton combined with various glycosidic residues, such as glucose, rhamnose, xylose, and arabinose. Among different carbohydrate hydrolases, β-glucosidase was considered the key enzyme for deglucosylated minor ginsenoside production (Song et al., 2017; Kim et al., 2022). Therefore, all 93 isolates from *P. bipinnatifidus* were preliminary screened for β-glucosidase activities by the agar selecting method, and 11 strains (belonging to 11 taxa, 8 genera) showed strong β-glucosidase activities. It is quite interesting that endophytic fungi isolated from *P. bipinnatifidus* represented a much higher percentage of strong β-glucosidase activities than those from the other two plants (*Epimedium sp.* and *Herminium monorchis*, in which saponins are not the main chemical constituents) in the same region (data not shown).

The biotransformation potential of the 11 isolates was examined with ginseng total saponins, and all strains presented activity of ginsenoside biotransformation with varied glycoside-hydrolyzing pathways. In view of literature reports, ginsenoside bioconversion by fungi with different origin is summarized in Table 4. It was found that transformation of protopanaxadiol-type ginsenoside Rb1/Rb2 (Ma et al., 2020; Kim et al., 2021) was mostly reported while transformation of protopanaxatriol-type ginsenoside Re by fungi was rare (Hong et al., 2011), probably due to the lack of microorganism resource with Re-specific glucosidase. Excitingly, two strains showed Re transformation activity in this study. In addition, the strain F8560, which was identified as *Penicillium citrinum*, was first found to have the ability to convert major ginsenoside Rb1 to minor ginsenoside CK. Further studies focusing on high efficiency production of CK with the enzyme from F8560 should be conducted since CK is one of the most valued minor ginsenosides, while the preparation of CK still remained costly (Ma et al., 2020; Kim et al., 2021). In addition, it is worth noting that three genera of *Ilyonectria*, *Sarocladium*, and *Lecanicillium* and all 11 taxa were first found to have the ability to transform ginsenosides in our study, which indicated that *P. bipinnatifidus* could be a new fungi resource with potent β-glucosidase for varied minor ginsenoside production.

**TABLE 4:** Summary of the transformation pathway for major ginsenosides by different fungi.

No	Taxa (accession number)	Transformation pathway	Reference
1	*Absidia coerulea*	Rg1→MT1; Rg1→F1	Dong et al. (2001) Liu et al. (2011)
2	*Arthrinium sp*	Rb→CK	Fu et al. (2016)
3	*Aspergillus niger*	Rg1→MT1; Rb1→Rd	Dong et al. (2001) Feng et al. (2016)
4	*Aspergillus ochraceus*	Rb1→Rd→F2; Rb2, Rc, Re→unidentified saponins	In this study
5	*Aspergillus pseudodeflectus*	Rb1→Rd→F2	In this study
6	*Aspergillus versicolor*	Rb1→Rd	Lin et al. (2015)
7	*Cladosporium fulvum*	Rb1→Rd	Zhao et al. (2009)
8	*Cladosporium oxysporum*	Rb1→Rd→F2	In this study
9	*Esteya vermicola*	Rb1→gypenoside LXXV	Hou et al. (2012)
10	*Fusarium sacchari*	Ginseng extracts→CK, C-Mx, G-Mc	Han et al. (2007)
11	*Fusarium solani*	Rb1→Rd→F2	In this study
12	*Ilyonectria robusta*	Rb1→Rd→F2; Rb2, Rc, Re→unidentified saponins	In this study
13	*Lecanicillium coprophilum*	Rb1→Rd	In this study
14	*Monascus pilosus*	Re→R13; Rg1→R22; Rh1→Rt4	Hong et al. (2011)
15	*Nodulisporium*	Rg1, F2, CK	Luo et al. (2013)
16	*Paecilomyces bainier*	Rb1→Rd	Ye et al. (2012)
17	*Penicillium citrinum*	Rb1→Rd→F2→CK	In this study
18	*Penicillium expansum*	Rb1→Rd	In this study
19	*Penicillum oxalicum*	Rb1→Rd; Rb1→Rd→F2→CK; Rb2→CO→CY→CK; Rc→Mb→Mc→CK; Rd→F2→CK	Gao et al. (2012)
			Gao et al. (2013)
			Gao et al. (2014)
20	*Penicillium rubens*	Rb1→Rd→F2; Rb2, Rc→unidentified saponins	In this study
21	*Penicillium sclerotiorum*	Rg1→F1	Zhao et al. (2009)
22	*Sarocladium bacillisporum*	Rb1→Rd	In this study
23	*Schizophyllum commune*	Rb1, Rc, Rb2, Rd→F2, CO, CY, CMc1, CMc, CK	Liu et al. (2019)
24	*Stereum hirsutum*	Rb1→F2→CK	Yang et al. (2021)
25	*Talaromyces variabilis*	Rb1→Rd→F2; Rb2, Rc→unidentified saponins	In this study
26	*Trichoderma longibrachiatum*	Rb1→Rd	Ge et al. (2015)

## Conclusion

In this study, the diversity, antimicrobial, and ginsenoside biotransformation potential of the endophytic fungi from *P. bipinnatifidus* were investigated for the first time. Our results illustrated that *P. bipinnatifidus* harbored abundant fungal endophytes resources, and a total of 93 representative morphotype strains were isolated and identified. Among 93 isolates, 26 strains exhibited inhibitory activities against at least one pathogenic bacterium or fungus, and 11 strains showed strong β-glucosidase activities and also presented with the ability of ginsenoside biotransformation with varied glycoside-hydrolyzing pathways. Excitingly, three genera, namely, *Ilyonectria*, *Sarocladium*, and *Lecanicillium*, and all 11 taxa were first found to have the ability to transform ginsenosides in our study. Overall, it was indicated that *P. bipinnatifidus* could be a new fungi resource with potential novel natural compounds with antimicrobial activity and potent β-glucosidase for varied minor ginsenoside production.

## Data Availability

The datasets presented in this study can be found in online repositories. The names of the repository/repositories and accession number(s) can be found in the article/Supplementary Material.
