# Supplementary material for: Diversity and Ginsenoside Biotransformation Potential of Cultivable Endophytic Fungi Associated With Panax bipinnatifidus var. bipinnatifidus in Qinling Mountains, China
Source: Front Pharmacol. 2022 Apr 4;13:762862. doi: 10.3389/fphar.2022.762862 (PMC9014171; doi:10.3389/fphar.2022.762862)
Supplement: Supplementary file 2 [file DataSheet1.PDF]

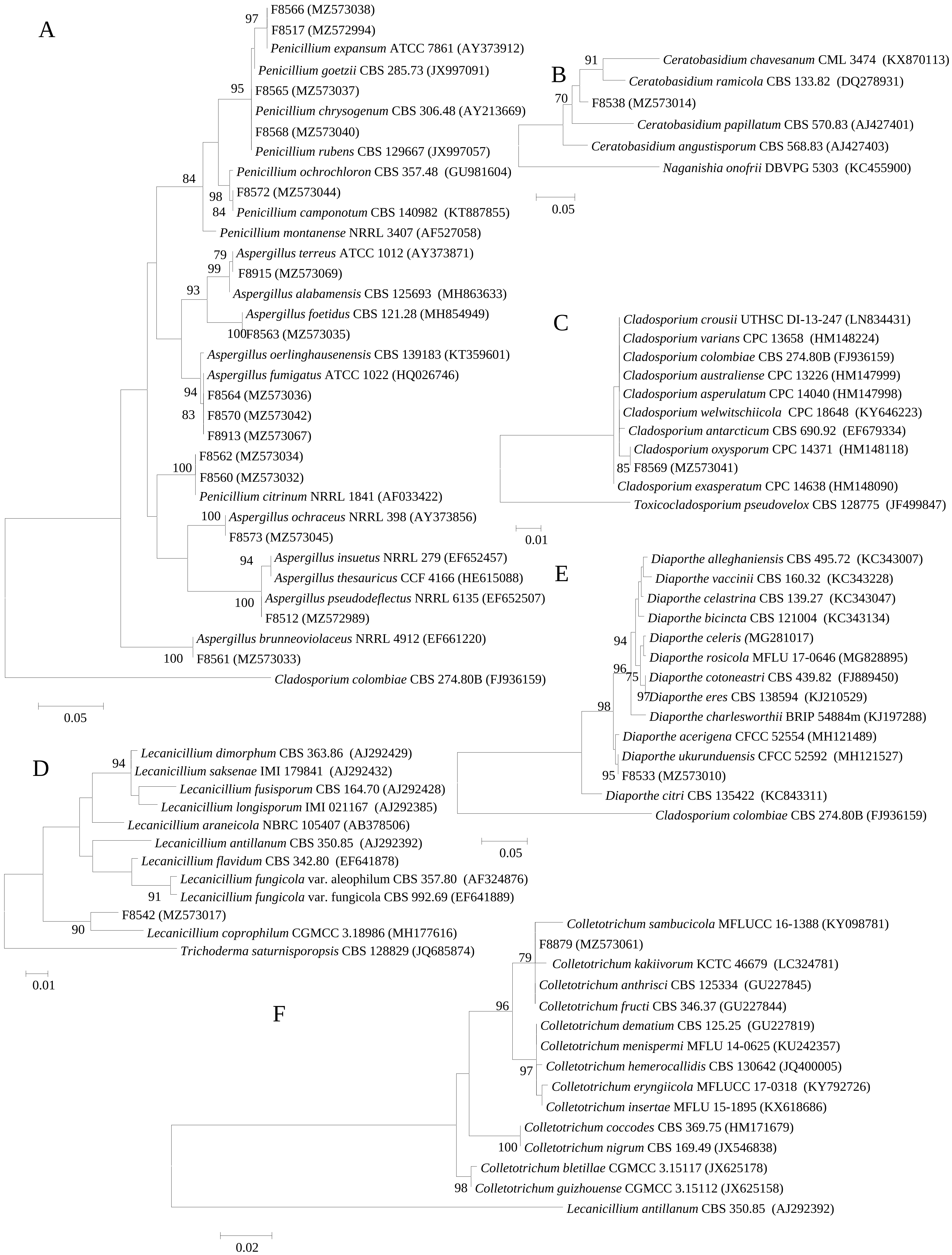

A
97
F8566 (MZ573038)
F8517 (MZ572994)
Penicillium expansum ATCC 7861 (AY373912)
Penicillium goetzii CBS 285.73 (JX997091)
95
F8565 (MZ573037)
Penicillium chrysogenum CBS 306.48 (AY213669)
F8568 (MZ573040)
Penicillium rubens CBS 129667 (JX997057)
84
Penicillium ochrochloron CBS 357.48 (GU981604)
98
F8572 (MZ573044)
84
Penicillium camponotum CBS 140982 (KT887855)
Penicillium montanense NRRL 3407 (AF527058)
79
Aspergillus terreus ATCC 1012 (AY373871)
99
F8915 (MZ573069)
93
Aspergillus alabamensis CBS 125693 (MH863633)
Aspergillus foetidus CBS 121.28 (MH854949)
100
F8563 (MZ573035)
Aspergillus oerlinghausenensis CBS 139183 (KT359601)
Aspergillus fumigatus ATCC 1022 (HQ026746)
94
F8564 (MZ573036)
83
F8570 (MZ573042)
F8913 (MZ573067)
100
F8562 (MZ573034)
F8560 (MZ573032)
Penicillium citrinum NRRL 1841 (AF033422)
100
Aspergillus ochraceus NRRL 398 (AY373856)
F8573 (MZ573045)
94
Aspergillus insuetus NRRL 279 (EF652457)
Aspergillus thesauricus CCF 4166 (HE615088)
100
Aspergillus pseudodeflectus NRRL 6135 (EF652507)
F8512 (MZ572989)
Aspergillus brunneoviolaceus NRRL 4912 (EF661220)
100
F8561 (MZ573033)
Cladosporium colombiae CBS 274.80B (FJ936159)
0.05
B
91
Ceratobasidium chavesanum CML 3474 (KX870113)
Ceratobasidium ramicola CBS 133.82 (DQ278931)
70
F8538 (MZ573014)
Ceratobasidium papillatum CBS 570.83 (AJ427401)
Ceratobasidium angustisporum CBS 568.83 (AJ427403)
Naganishia onofrii DBVPG 5303 (KC455900)
0.05
C
Cladosporium crousii UTHSC DI-13-247 (LN834431)
Cladosporium varians CPC 13658 (HM148224)
Cladosporium colombiae CBS 274.80B (FJ936159)
Cladosporium australiense CPC 13226 (HM147999)
Cladosporium asperulatum CPC 14040 (HM147998)
Cladosporium welwitschiicola CPC 18648 (KY646223)
Cladosporium antarcticum CBS 690.92 (EF679334)
Cladosporium oxysporum CPC 14371 (HM148118)
85
F8569 (MZ573041)
Cladosporium exasperatum CPC 14638 (HM148090)
Toxicocladosporium pseudovelox CBS 128775 (JF499847)
0.01
E
Diaporthe alleghaniensis CBS 495.72 (KC343007)
Diaporthe vaccinii CBS 160.32 (KC343228)
Diaporthe celastrina CBS 139.27 (KC343047)
Diaporthe bicincta CBS 121004 (KC343134)
94
Diaporthe celeris (MG281017)
Diaporthe rosicola MFLU 17-0646 (MG828895)
96
75
Diaporthe cotoneastri CBS 439.82 (FJ889450)
97
Diaporthe eres CBS 138594 (KJ210529)
98
Diaporthe charlesworthii BRIP 54884m (KJ197288)
Diaporthe acerigena CFCC 52554 (MH121489)
Diaporthe ukurunduensis CFCC 52592 (MH121527)
95
F8533 (MZ573010)
Diaporthe citri CBS 135422 (KC843311)
Cladosporium colombiae CBS 274.80B (FJ936159)
0.05
D
94
Lecanicillium dimorphum CBS 363.86 (AJ292429)
Lecanicillium saksenae IMI 179841 (AJ292432)
Lecanicillium fusisporum CBS 164.70 (AJ292428)
Lecanicillium longisporum IMI 021167 (AJ292385)
Lecanicillium araneicola NBRC 105407 (AB378506)
Lecanicillium antillanum CBS 350.85 (AJ292392)
Lecanicillium flavidum CBS 342.80 (EF641878)
Lecanicillium fungicola var. aleophilum CBS 357.80 (AF324876)
91
Lecanicillium fungicola var. fungicola CBS 992.69 (EF641889)
F8542 (MZ573017)
90
Lecanicillium coprophilum CGMCC 3.18986 (MH177616)
Trichoderma saturnisporopsis CBS 128829 (JQ685874)
0.01
F
Colletotrichum sambucicola MFLUCC 16-1388 (KY098781)
F8879 (MZ573061)
79
Colletotrichum kakiivorum KCTC 46679 (LC324781)
Colletotrichum anthrisci CBS 125334 (GU227845)
Colletotrichum fructi CBS 346.37 (GU227844)
96
Colletotrichum dematium CBS 125.25 (GU227819)
Colletotrichum menispermi MFLU 14-0625 (KU242357)
Colletotrichum hemerocallidis CBS 130642 (JQ400005)
97
Colletotrichum eryngiicola MFLUCC 17-0318 (KY792726)
Colletotrichum insertae MFLU 15-1895 (KX618686)
Colletotrichum coccodes CBS 369.75 (HM171679)
100
Colletotrichum nigrum CBS 169.49 (JX546838)
Colletotrichum bletillae CGMCC 3.15117 (JX625178)
98
Colletotrichum guizhouense CGMCC 3.15112 (JX625158)
Lecanicillium antillanum CBS 350.85 (AJ292392)
0.02

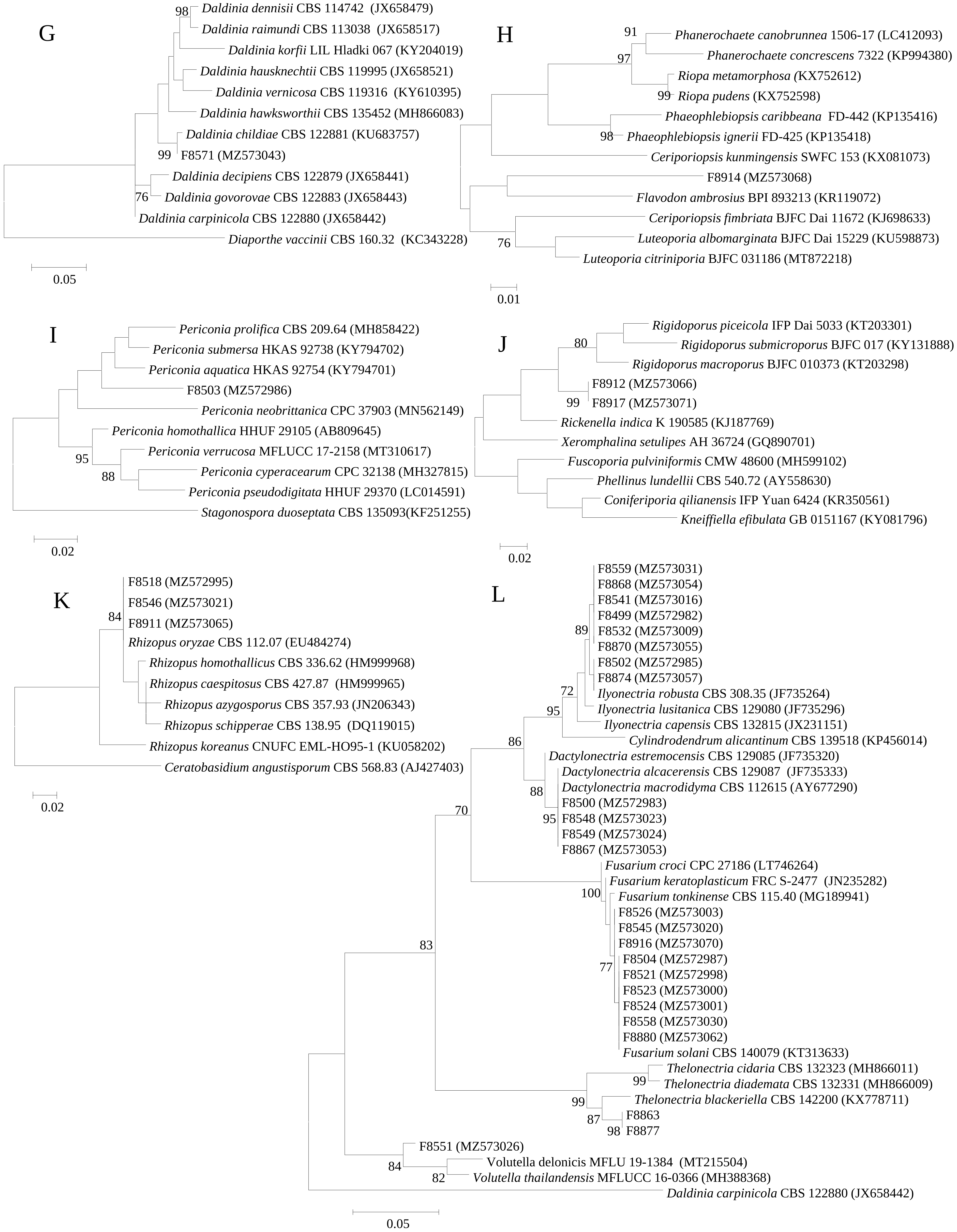
G
98
Daldinia dennisii CBS 114742 (JX658479)
Daldinia raimundi CBS 113038 (JX658517)
Daldinia korfii LIL Hladki 067 (KY204019)
Daldinia hausknechtii CBS 119995 (JX658521)
Daldinia vernicosa CBS 119316 (KY610395)
Daldinia hawksworthii CBS 135452 (MH866083)
Daldinia childiae CBS 122881 (KU683757)
99
F8571 (MZ573043)
Daldinia decipiens CBS 122879 (JX658441)
76
Daldinia govorovae CBS 122883 (JX658443)
Daldinia carpinicola CBS 122880 (JX658442)
Diaporthe vaccinii CBS 160.32 (KC343228)
0.05
H
91
Phanerochaete canobrunnea 1506-17 (LC412093)
97
Phanerochaete concrescens 7322 (KP994380)
Riopa metamorphosa (KX752612)
99
Riopa pudens (KX752598)
Phaeophlebiopsis caribbeana FD-442 (KP135416)
98
Phaeophlebiopsis ignerii FD-425 (KP135418)
Ceriporiopsis kunmingensis SWFC 153 (KX081073)
F8914 (MZ573068)
Flavodon ambrosius BPI 893213 (KR119072)
Ceriporiopsis fimbriata BJFC Dai 11672 (KJ698633)
Luteoporia albomarginata BJFC Dai 15229 (KU598873)
76
Luteoporia citriniporia BJFC 031186 (MT872218)
0.01
I
Periconia prolifica CBS 209.64 (MH858422)
Periconia submersa HKAS 92738 (KY794702)
Periconia aquatica HKAS 92754 (KY794701)
F8503 (MZ572986)
Periconia neobrittanica CPC 37903 (MN562149)
Periconia homothallica HHUF 29105 (AB809645)
95
Periconia verrucosa MFLUCC 17-2158 (MT310617)
88
Periconia cyperacearum CPC 32138 (MH327815)
Periconia pseudodigitata HHUF 29370 (LC014591)
Stagonospora duoseptata CBS 135093(KF251255)
0.02
J
Rigidoporus piceicola IFP Dai 5033 (KT203301)
80
Rigidoporus submicroporus BJFC 017 (KY131888)
Rigidoporus macroporus BJFC 010373 (KT203298)
F8912 (MZ573066)
99
F8917 (MZ573071)
Rickenella indica K 190585 (KJ187769)
Xeromphalina setulipes AH 36724 (GQ890701)
Fuscoporia pulviniformis CMW 48600 (MH599102)
Phellinus lundellii CBS 540.72 (AY558630)
Coniferiporia qilianensis IFP Yuan 6424 (KR350561)
Kneiffiella efibulata GB 0151167 (KY081796)
0.02
K
F8518 (MZ572995)
F8546 (MZ573021)
84
F8911 (MZ573065)
Rhizopus oryzae CBS 112.07 (EU484274)
Rhizopus homothallicus CBS 336.62 (HM999968)
Rhizopus caespitosus CBS 427.87 (HM999965)
Rhizopus azygosporus CBS 357.93 (JN206343)
Rhizopus schipperae CBS 138.95 (DQ119015)
Rhizopus koreanus CNUFC EML-HO95-1 (KU058202)
Ceratobasidium angustisporum CBS 568.83 (AJ427403)
0.02
L
F8559 (MZ573031)
F8868 (MZ573054)
F8541 (MZ573016)
F8499 (MZ572982)
89
F8532 (MZ573009)
F8870 (MZ573055)
F8502 (MZ572985)
F8874 (MZ573057)
72
Ilyonectria robusta CBS 308.35 (JF735264)
95
Ilyonectria lusitanica CBS 129080 (JF735296)
Ilyonectria capensis CBS 132815 (JX231151)
86
Cylindrodendrum alicantinum CBS 139518 (KP456014)
Dactylonectria estremocensis CBS 129085 (JF735320)
Dactylonectria alcacerensis CBS 129087 (JF735333)
88
Dactylonectria macrodidyma CBS 112615 (AY677290)
F8500 (MZ572983)
70
95
F8548 (MZ573023)
F8549 (MZ573024)
F8867 (MZ573053)
Fusarium croci CPC 27186 (LT746264)
Fusarium keratoplasticum FRC S-2477 (JN235282)
100
Fusarium tonkinense CBS 115.40 (MG189941)
F8526 (MZ573003)
F8545 (MZ573020)
83
F8916 (MZ573070)
F8504 (MZ572987)
77
F8521 (MZ572998)
F8523 (MZ573000)
F8524 (MZ573001)
F8558 (MZ573030)
F8880 (MZ573062)
Fusarium solani CBS 140079 (KT313633)
Thelonectria cidaria CBS 132323 (MH866011)
99
Thelonectria diademata CBS 132331 (MH866009)
99
Thelonectria blackeriella CBS 142200 (KX778711)
87
F8863
98
F8877
F8551 (MZ573026)
Volutella delonicis MFLU 19-1384 (MT215504)
84
82
Volutella thailandensis MFLUCC 16-0366 (MH388368)
Daldinia carpinicola CBS 122880 (JX658442)
0.05

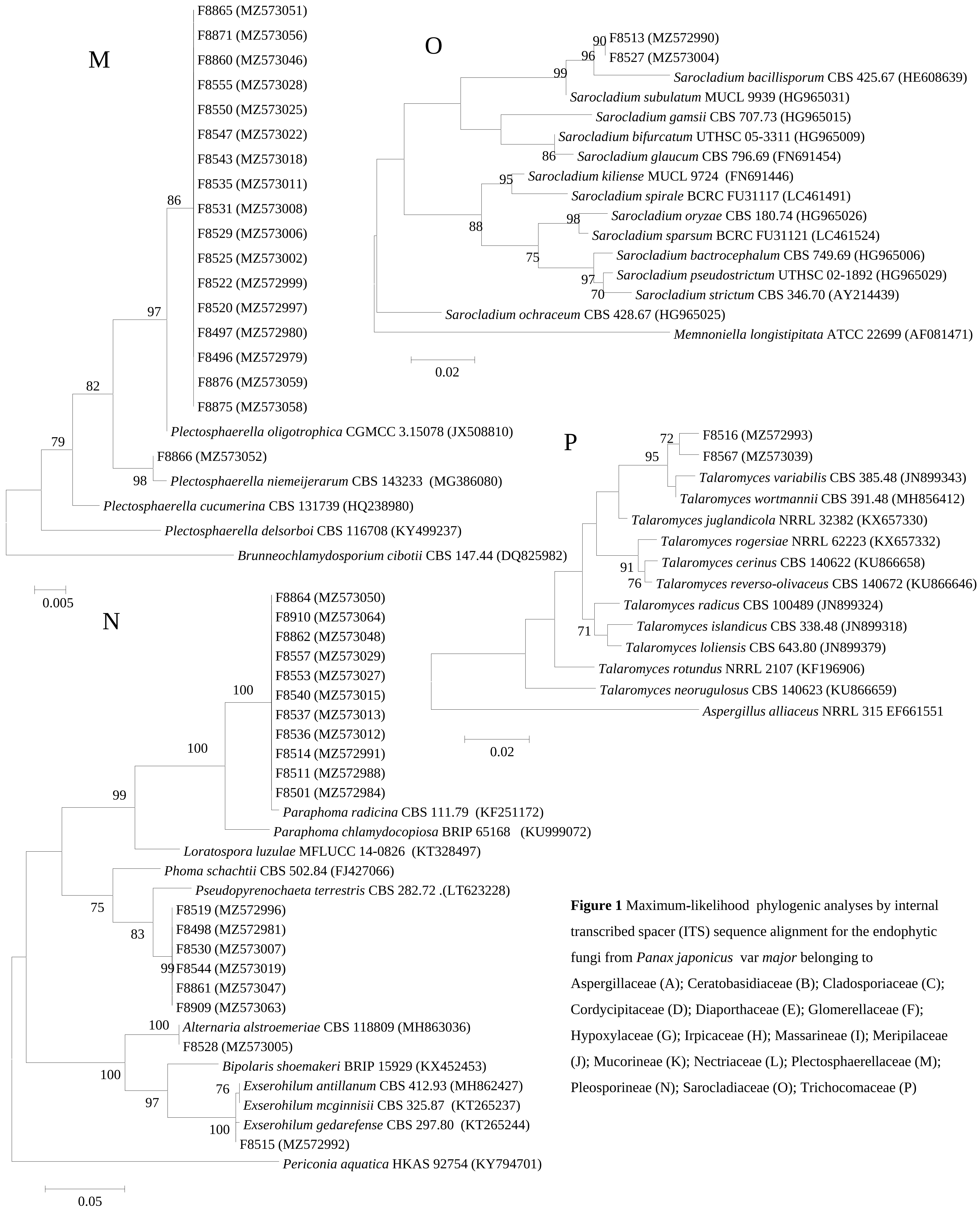

Figure 1 Maximum-likelihood phylogenic analyses by internal transcribed spacer (ITS) sequence alignment for the endophytic fungi from *Panax japonicus* var *major* belonging to Aspergillaceae (A); Ceratobasidiaceae (B); Cladosporiaceae (C); Cordycipitaceae (D); Diaporthaceae (E); Glomerellaceae (F); Hypoxylaceae (G); Irpicaceae (H); Massarineae (I); Meripilaceae (J); Mucorineae (K); Nectriaceae (L); Plectosphaerellaceae (M); Pleosporineae (N); Sarocladiaceae (O); Trichocomaceae (P)
